# Comparison of the Novel Thrombolytic Constitutively Active ADAMTS13 With Clinical Thrombolytics in a Murine Stroke Model

**DOI:** 10.1161/STROKEAHA.125.050848

**Published:** 2025-04-02

**Authors:** Lucy Roberts, Graham Coutts, Ben R. Dickie, Craig J. Smith, Kieron South, Stuart M. Allan

**Affiliations:** Division of Neuroscience, School of Biological Sciences (L.R., G.C., K.S., S.M.A.), Faculty of Biology, Medicine and Health, Manchester Academic Health Science Centre, The University of Manchester, United Kingdom.; Division of Informatics, Imaging and Data Sciences, School of Health Sciences (B.R.D.), Faculty of Biology, Medicine and Health, Manchester Academic Health Science Centre, The University of Manchester, United Kingdom.; Division of Cardiovascular Sciences, School of Medical Sciences (C.J.S.), Faculty of Biology, Medicine and Health, Manchester Academic Health Science Centre, The University of Manchester, United Kingdom.; Geoffrey Jefferson Brain Research Centre, Manchester Academic Health Science Centre, Northern Care Alliance NHS Foundation Trust, The University of Manchester, United Kingdom (L.R., G.C., B.R.D., C.J.S., K.S., S.M.A.).; Manchester Centre for Clinical Neurosciences, Manchester Academic Health Science Centre, Salford Royal NHS Foundation Trust, United Kingdom (C.J.S.).

**Keywords:** cardiovascular diseases, cerebral hemorrhage, fibrinolytic agents, ischemic stroke, therapeutics, thrombolytic therapy

## Abstract

**BACKGROUND:**

r-tPA (recombinant tissue-type plasminogen activator) and its variant, TNK (tenecteplase), are the currently approved thrombolytic drugs for the treatment of acute ischemic stroke, but they are ineffective in a proportion of patients due to r-tPA resistance of platelet-rich thrombi. A novel thrombolytic, caADAMTS13 (constitutively active a disintegrin and metalloproteinase with a thrombospondin type 1 motif, member 13) has been shown to improve experimental stroke outcomes where platelet-rich thrombi are present but have not been directly compared with r-tPA or TNK.

**METHODS:**

We conducted a direct comparison of caADAMTS13 versus r-tPA versus TNK versus vehicle control in the ferric chloride–mediated distal middle cerebral artery occlusion model in mice, which features platelet and VWF (von Willebrand Factor)–rich thrombi that reproduce r-tPA-resistant occlusion. Treatments were administered intravenously 1 hour after ferric chloride application by bolus injection or bolus followed by infusion, as translationally applicable. Laser speckle contrast imaging measured early reperfusion over the hour following treatment, and magnetic resonance imaging measured cerebral blood flow and lesion volume at 24 hours.

**RESULTS:**

Reperfusion 1 hour after treatment was greatest in caADAMTS13-treated animals. Later cerebral blood flow, 24 hours post-treatment, within the stroke-affected hypoperfused area was higher in caADAMTS13 and r-tPA but not TNK-treated mice. Functionally, this led to the absence of an initial behavioral deficit in caADAMTS13-treated mice, alongside a smaller lesion volume at 24 hours and reduced extent of bleeding.

**CONCLUSIONS:**

These findings demonstrate an overall suggestion that caADAMTS13 has improved thrombolytic efficacy, compared with current stroke treatments, against platelet-rich thrombi, for which there is currently an unmet clinical need.

The therapeutic aim of thrombolysis in acute ischemic stroke (AIS) is the fast and effective removal of thrombi occluding cerebral arteries to achieve reperfusion.^1^ For many years, the only clinically approved thrombolytic to achieve reperfusion has been r-tPA (recombinant tissue-type plasminogen activator; alteplase). However, r-tPA produces effective recanalization in as few as 10% to 35% of patients, depending on the population and cause studied, and is associated with a significant risk of intracerebral hemorrhage.^2,3^ More recently, there has been an increase in recommendations for the use of TNK (tenecteplase) as a thrombolytic for AIS.^4^ TNK is a r-tPA variant with increased fibrin specificity and increased resistance to inhibition and, thus, has a longer half-life than r-tPA.^5^ This enables TNK administration as a bolus, as opposed to the administration of r-tPA as a 10% volume bolus followed by 90% volume infusion, and a reduction in the required therapeutic dose. Clinical studies have demonstrated that 0.25-mg/kg TNK has a comparable safety profile to 0.9-mg/kg r-tPA in terms of hemorrhagic transformation events and is at least as effective in terms of reperfusion and functional outcomes.^2,6–9^ In preclinical AIS, r-tPA and TNK both have similar efficacy^10,11^ with increased safety of TNK only at early administration.^10^

The comparable efficacy and shared mechanism of action may suggest that the phenomenon of r-tPA resistance, where r-tPA is ineffective at breaking down altered fibrin and nonfibrinous structural components, may similarly occur with TNK. AIS thrombi are broadly characterized into 2 compositions: red blood cell–rich consisting of areas packed with red blood cells within a thin, loose meshwork of fibrin, and platelet-rich consisting of dense fibrin and VWF (von Willebrand Factor) structures that delineate platelet-filled areas.^12,13^ It is the loose fibrin meshwork of the red blood cell–rich thrombus composition that plasmin derived from r-tPA activation is effective at degrading, while the platelet-rich composition is r-tPA-resistant. The greater the proportion of platelet and VWF components, the greater the r-tPA resistance of thrombi.^14^

Several preclinical studies have investigated targeting VWF as an alternative thrombolytic strategy, including utilizing the endogenous VWF regulator, ADAMTS13 (a disintegrin and metalloproteinase with a thrombospondin type 1 motif, member 13). Targeting the VWF/ADAMTS13 axis is protective in AIS,^13,15–19^ but the efficacy of wild-type ADAMTS13 is limited due to its conformational quiescence.^17,20^ Inducing point mutations in the linker 3 region of ADAMTS13 produced a structural mutation that formed caADAMTS13 (constitutively active ADAMTS13), which, in 2 preclinical AIS models, demonstrated an ability to improve cerebral blood flow, reduce lesion volume, reduce platelet and fibrin deposition, and displayed anti-inflammatory properties.^21^ caADAMTS13 additionally displayed antithromboinflammatory properties in vitro and in vivo by reduction of neutrophil rolling and migration.^22^ For further preclinical translational development of novel thrombolytics, a head-to-head comparison against established thrombolytics should be conducted.^1^ Therefore, the aim of this study was to perform a direct comparison between the novel AIS thrombolytic, caADAMTS13, the thrombolytics in current clinical practice, r-tPA and TNK, and their respective vehicle controls in a murine model of thromboembolic stroke.

## Methods

The data presented are available from the corresponding author upon reasonable request. Full method descriptions are available in the Supplemental Material. Animal procedures were carried out in accordance with the UK Animal Scientific Procedures Act (1986), approved by the local ethical review board (The University of Manchester), and followed ARRIVE guidelines (Animal Research: Reporting of In Vivo Experiments; Supplemental Material).^23^ Male (31–40 g) and (26–38 g) female CD1 mice (Charles River, United Kingdom) were used (1:1 ratio within treatment groups). Sample sizes were calculated to allow for adequate statistical power based on previous data using a significance level of α=0.05 with 80% power to detect significant differences. Animals were electronically randomly assigned to the experiment, and treatments were randomized within the sexes, with researchers blinded to treatment. Blinding was maintained throughout experimentation and data analysis. To ensure allocation concealment, sequentially labeled tubes of unidentifiable treatment materials were used post-occlusion, at the time of treatment. While it was not practically feasible to blind allocation to bolus or infusion administration methods, the treatments and respective vehicle controls were visually indistinguishable and included across all administration routes.

**Figure 1.**
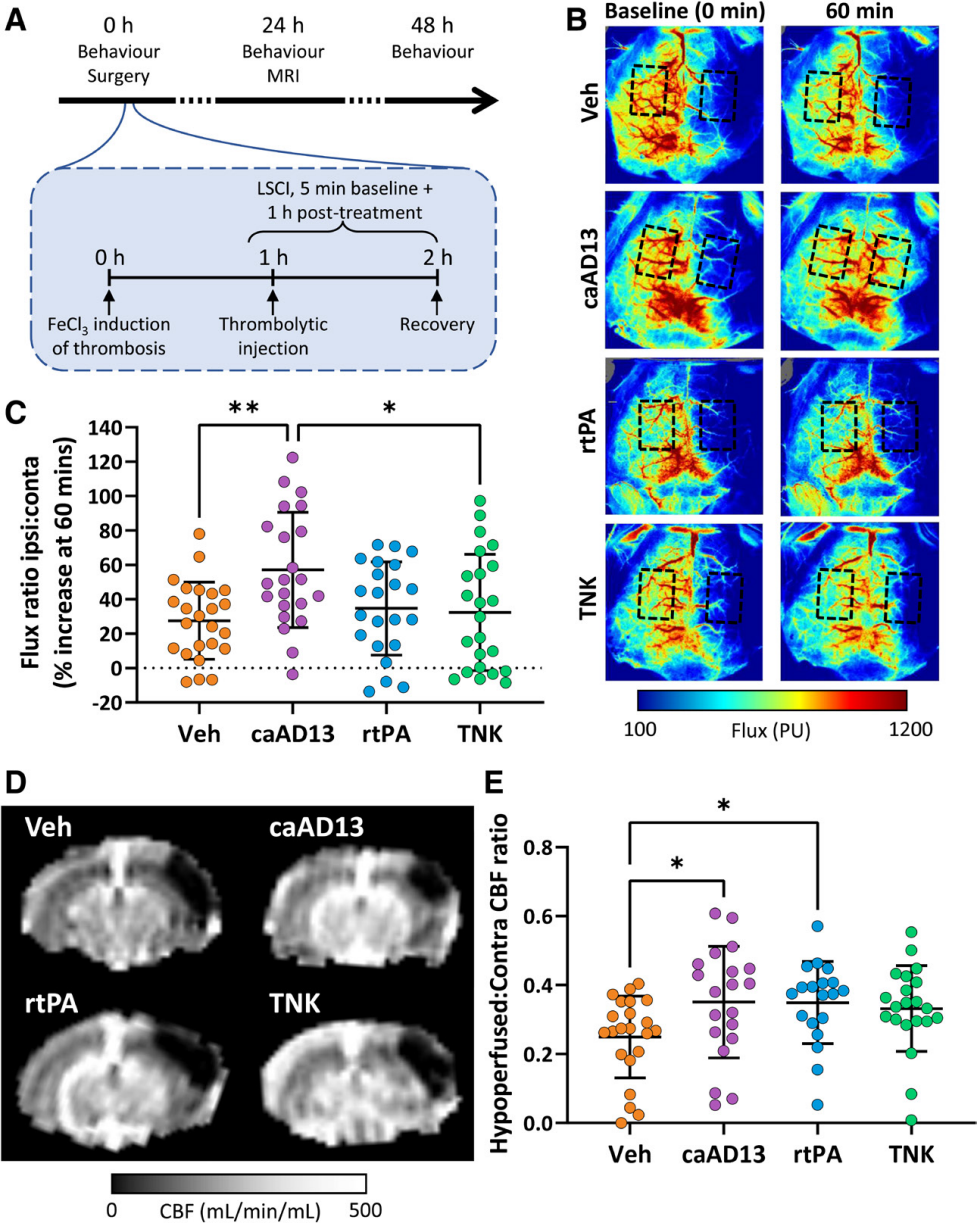
**Improvement of cerebral blood flow to the stroke-affected area at both 1 and 24 hours following caADAMTS13 (constitutively active a disintegrin and metalloproteinase with a thrombospondin type 1 motif, member 13) administration. A**, Acute ischemic stroke was induced by topical ferric chloride application on the middle cerebral artery (MCA), and treatments were administered starting 1 hour poststroke induction and laser speckle contrast imaging (LSCI) conducted at 1-minute intervals for 5 minutes pre-treatment and 1 hour post-treatment. Behavior (28-point neuroscore) was conducted at baseline (0 hour), 24 hours, and 48 hours poststroke. Magnetic resonance imaging (MRI) was conducted at 24 hours poststroke with arterial sin labeling MRI to determine cerebral blood flow and high-spatial-resolution T2-weighted MRI to quantify lesion volume. **B**, Representative LSCI of reperfusion from each treatment group at baseline pre-injection and 60 min after treatment injection. Regions of interest for quantification over the ipsilateral and contralateral hemispheres are indicated (black, dashed rectangles). **C**, Ratios of the ipsilateral to contralateral hemisphere flux values were calculated, and the percentage flux ratio between the baseline values pre-treatment and 60 minutes post-treatment was determined (combined sex, n=22–24; male, n=11–12; and female, n=11–12). **D**, Representative MRI arterial spin labeling images from an animal in each treatment group for quantification of cerebral blood flow (CBF). **E**, Ratios of the CBF within the hypoperfused (stroke) area in the ipsilateral hemisphere to the CBF within the whole of the contralateral hemisphere were calculated for individual animals (combined sex, n=19–22; male, n=10–11; and female, n=9–11). Error bars represent mean±SD. Statistics were performed using a 1-way ANOVA followed by (**C**) the Tukey and (**E**) Dunn multiple comparisons post hoc tests. *P<0.05; **P<0.01. FeCl_3_ indicates ferric chloride; caAD13, caADAMTS13; r-tPA, recombinant tissue-type plasminogen activator; TNK, tenecteplase; and Veh, vehicle control.

The experimental design overview is presented in Figure 1A. An intravenous administration route was established by a tail vein catheter. Ferric chloride (FeCl_3_)–mediated distal middle cerebral artery occlusion (dMCAo), which forms platelet and VWF-rich, r-tPA-resistant thrombi, was performed using previously described surgical techniques,^24^ performed under inhaled isoflurane (1.5%–2.5% in 30%/70% O_2_/NO_2_) with temperature controlled using a feedback heating pad (37 °C±0.5 °C). Two rounds of 5-minute, 20% (w/v) FeCl_3_ applications were conducted, with saline wash between. A priori exclusions pre-treatment included MCA damage, failure to establish a viable tail vein administration route, and insufficient reduction in cerebral reperfusion based on baseline laser speckle contrast imaging.

Laser speckle contrast imaging, the primary outcome measure for cerebral reperfusion, was performed every 1 minute, for 5 minutes pre-injection, and 1 hour post-injection as previously described.^21^ Treatments were administered by intravenous tail vein injection, starting at 1 hour post-FeCl_3_ application. caADAMTS13 (6 mg/kg),^21^ TNK (2.5 mg/kg),^10^ and their respective vehicle controls were administered as a bolus. r-tPA (10 mg/kg)^10,18,19^ and its respective vehicle control were administered as a 10% volume bolus followed by a 90% volume infusion over 40 minutes. A priori exclusions post-treatment included failure to administer full treatment volume and failure to recover to an acceptable condition.

Neurological deficits were assessed using a 28-point neuroscore^25^ at baseline, 24 hours, and 48 hours post-surgery. Twenty-four hours after treatment, arterial spin labeling magnetic resonance imaging (MRI) was used to assess cerebral blood flow, and high-spatial-resolution T2-weighted MRI was used to assess lesion volume. MRI was performed under inhaled isoflurane (1.5%–2%), and the core temperature was maintained at 37 °C with a feedback-controlled fan heater and rectal probe. Detailed MRI sequence methods can be found in the Supplemental Material. Cryopreserved, 10-µm coronal brain sections, sampled at 500-µm intervals throughout the brain, were stained with hematoxylin and eosin for quantification of hemorrhagic transformation with all stained sections considered for hemorrhage quantification. Data are presented as mean±SD. For statistical comparisons, groups were tested for normality, and all groups were compared with each other.

## Results

There were no differences between the treatment groups’ respective vehicle controls; consequently, vehicle treatments were considered as a single group. No significant sex differences were identified throughout the study; therefore, mixed-sex groups were investigated. All treated animals were included in each measurement, except in the case of mortality (5 animals), including due to anesthesia, not recovering to an acceptable condition or displaying severe stroke symptoms^26^ (Figure S1), or where technical difficulties arose leading to uncollected data.

**Figure 2.**
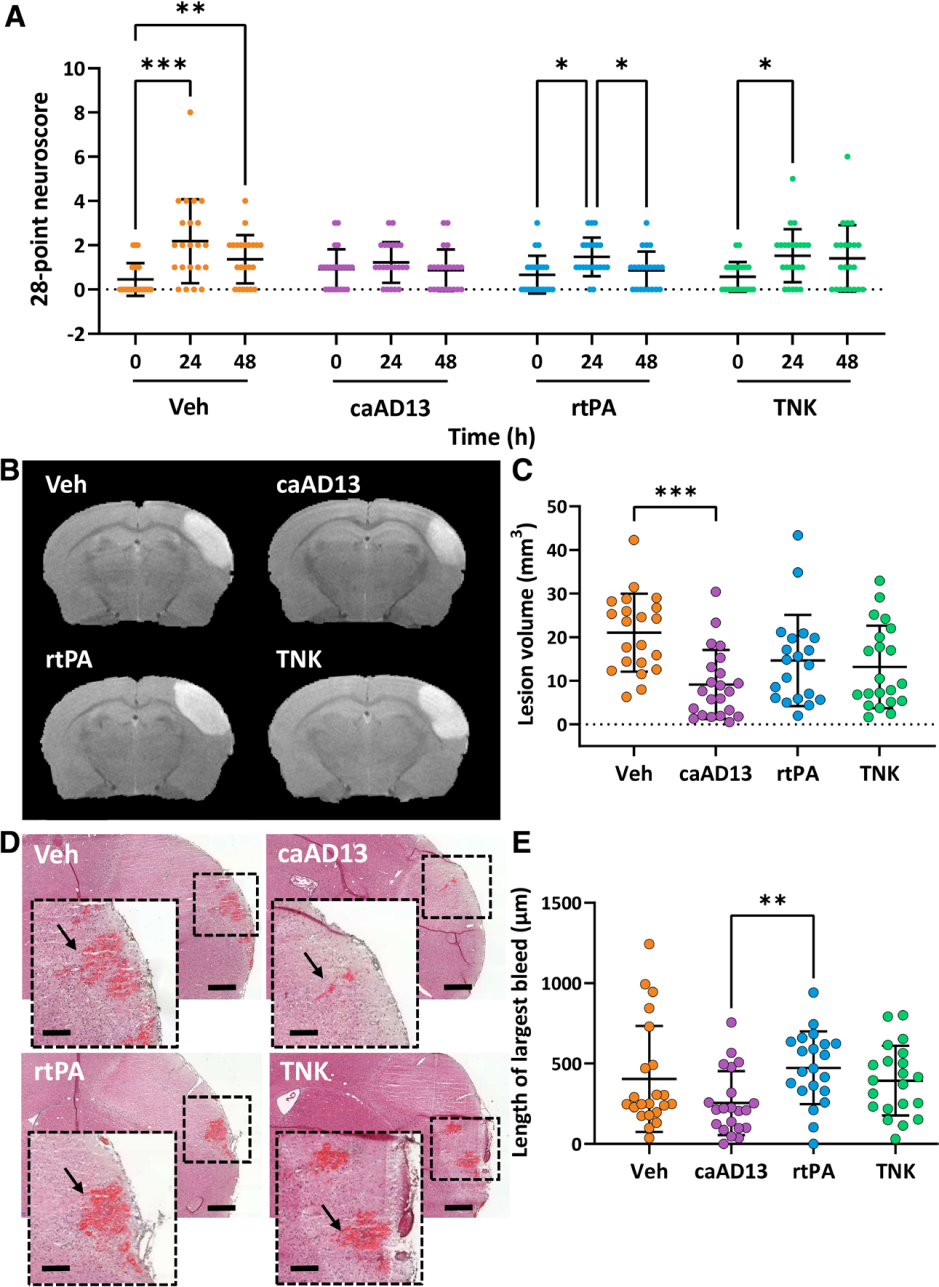
**caADAMTS13 (constitutively active a disintegrin and metalloproteinase with a thrombospondin type 1 motif, member 13) thrombolysis 1 hour post-acute ischemic stroke prevented behavioral deficits and reduced lesion volume at 24 hours. A**, A 28-point neuroscore test was conducted at baseline (0 hour), 24 hours, and 48 hours poststroke (combined sex, n=21–23; male, n=11–12; and female, n=10–12). **B**, Representative T2-magnetic resonance (MR) images from an animal in each of the treatment groups, 24 hours poststroke. The lesion is visible as the hyperintense area in the right-hand cortex. **C**, Lesion volumes were quantified from the T2-MR images by measuring the area of hyperintensity (combined sex, n=19–22; male, n=11; and female, n=9–11). **D**, Representative hematoxylin and eosin stained brain sections from an animal in each treatment group, 48 hours poststroke. Error bars are 500 µm for the lower magnification image and 200 µm for the higher magnification insert. Bleeds are visible as the red-stained area and are indicated by the arrow in the insert. **E**, The maximum length of the largest hemorrhage for each mouse was quantified (combined sex, n=19–21; male, n=8–11; and female, n=9–10). Error bars represent mean±SD. Statistics were performed using (**A**) a mixed-effects analysis followed by the Tukey multiple comparisons post hoc test and (**C** and **E**) a Kruskal-Wallis test followed by the Dunn multiple comparisons post hoc test. *P<0.05; **P<0.01; and ***P<0.001. caAD13 indicates caADAMTS13; r-tPA, recombinant tissue-type plasminogen activator; TNK, tenecteplase; and veh, vehicle control.

Early reperfusion, measured by the increase in ipsilateral:contralateral ratio laser speckle contrast imaging perfusion 1 hour after thrombolysis, was 2.1-fold greater (*P*=0.006) with caADAMTS13 treatment than vehicle control. Early reperfusion by laser speckle contrast imaging in the caADAMTS13 treatment group was additionally 1.8-fold greater (*P*=0.031) than TNK and 1.6-fold greater (*P*=0.065) than r-tPA. Comparisons between the vehicle control, r-tPA, and TNK groups were not significantly different (Figure 1B and 1C; Figure S2). At a later timepoint, 24 hours, cerebral blood flow within the hypoperfused area of the ipsilateral hemisphere, measured by arterial spin labeling MRI, was 41% (*P*=0.037) and 40% (*P*=0.045) greater in the caADAMTS13- and r-tPA-treated mice, respectively, compared with control (Figure 1D and 1E). Functionally, r-tPA-, TNK-, and vehicle-treated mice suffered significant neurological deficits 24 hours poststroke compared with their 0-hour baseline score, whereas caADAMTS13-treated mice did not incur this initial deficit (Figure 2A). However, between-treatment-group comparisons revealed no significant differences in neurological deficits. A 56% decrease (*P*=0.0003) in the resulting lesion volume compared with the vehicle, quantified at 24 hours by MRI, was observed in the caADAMTS13 treatment group (Figure 2B and 2C). The spatial extent of bleeds was lower in the caADAMTS13 group compared with r-tPA (Figure 2D and 2E), with bleed length being independent of lesion volume (Table S1).

## Discussion

The improvement in reperfusion following caADAMTS13 treatment with respect to the vehicle control reproduces previous results.^21^ Some spontaneous reperfusion occurred in all treatment groups, including with vehicle treatment, which may occur due to variability in the size of the occlusive thrombi formed^13^ and is comparable to that previously observed.^10,13,18,19,21^ Between the early (1 hour) and later (24 hours) reperfusion time points, there appeared to be a relative improvement in the r-tPA and TNK groups’ reperfusion compared with the vehicle and caADAMTS13 treatment groups. This might be expected as caADAMTS13 already had a significant effect on reperfusion within the first hour of treatment; therefore, the incremental change from 1 to 24 hours will be less for the caADAMTS13 group compared with r-tPA and TNK, which show no early reperfusion effect. As proposed elsewhere, this later improvement in cerebral blood flow suggests that r-tPA, and by extension possibly TNK, may be triggering partial reperfusion at a time point between the first and second measurements (1–24 hours).^19^ Nevertheless, while the aim of AIS treatment is to quickly and effectively recanalize and reperfuse occluded blood vessels to prevent irretrievable loss of neuronal tissue and improve stroke outcomes, earlier reperfusion, as observed only with caADAMTS13, tended to display advantages in terms of reducing lesion volume, reducing bleeding events, and reducing initial neurological deficit.

The success of cerebral reperfusion is dependent on thrombus composition. FeCl_3_ dMCAo was used due to its formation of platelet- and VWF-rich, fibrin-poor thrombi^13,18^ that are largely resistant to r-tPA, albeit alongside a slight trend to improvement following r-tPA treatment,^18,19^ consistent with the findings here. r-tPA’s variant, TNK, had comparable effects on stroke outcome to r-tPA in our hands, which also holds true for lesion volume in a fibrin-rich dMCAo model.^10^ As anticipated, caADAMTS13 exhibited improved, or trends to improved, efficacy against platelet- and VWF-rich thrombi, a composition for which there is currently an unmet need for clinical thrombolytics.^14^ VWF is present in all thrombi to varying proportions^13,14^; therefore, targeting VWF with caADAMTS13 could additionally be effective against VWF components within platelet-rich and mixed composition thrombi.^12^ Previous studies have investigated means of targeting fibrin and VWF components in parallel, for example, Microlyse, a thrombolytic that localizes plasminogen activation to VWF, with subsequent plasmin cleavage of VWF.^19,27^ While caADAMTS13 is suggested to enhance fibrinolysis in vitro,^21^ this has not been demonstrated in vivo. Future studies should assess caADAMTS13 in fibrin-rich dMCAo, as the potential for a thrombolytic therapy that can act via 2 major thrombus components may significantly increase the pool of patients with AIS for whom thrombolytic therapy may be effective.

This study has important limitations to consider. Although we found no evidence of sex differences, this study represents the first time that caADAMTS13, and to the best of our knowledge, r-tPA and TNK have been tested as thrombolytics in both sexes in FeCl_3_ dMCAo. The absence of observed sex differences in a young, noncomorbid population should not be overinterpreted, and sex differences should continue to be considered alongside comorbidities and additional complexities in models to further enhance translation beyond the scope of the current study.^28^ While it is argued that neurological deficit can predict the outcomes of patients with AIS, several other behavioral tests could provide greater nuance by assessing specific motor, cognitive, and sensory functions.^29^ Moreover, although this study focused on the acute perfusion effects of the thrombolytics, future studies incorporating behavioral testing over an extended timeframe could provide a better prediction of the functional significance of the early reperfusion achieved by caADAMTS13. Finally, thrombolytic safety assessment by hemorrhage bleed length quantification may not accurately reflect bleeding extent, possibly underestimating or overestimating some bleeds due to 3-dimensional morphology. Currently, all instances of bleeding are considered, but the anterior-posterior distance between brain sections is large in comparison to potential bleed sizes. Sampling sections with higher frequency could enable accurate volumetric quantification of the hemorrhage. In conjunction, implementing a classification system for assessing hemorrhagic transformation characteristics, like those implemented elsewhere,^30,31^ could enhance the qualitative assessment of bleeds, as would employment of T2*-MRI for quantification.

## Conclusions

Overall, the results suggest that caADAMTS13 may provide improved early thrombolytic properties compared with the standard of care against platelet- and VWF-rich thrombi in a preclinical stroke model. The early reperfusion achieved by caADAMTS13, rather than later reperfusion also suggested by r-tPA and TNK, led to the trend for a greater extent of improved stroke outcomes of caADAMTS13 over r-tPA and TNK.

## ARTICLE INFORMATION

### Sources of Funding

This study was supported by the British Heart Foundation 4-Year PhD Studentship Program (grant FS/18/62/34183) to Dr Roberts and The University of Manchester Innovation Factory and a Stroke Association Non-Clinical Lectureship Award to Dr South (grant SA NCL 22100001).

### Disclosures

The Ala1144Val ADAMTS13 variant (caADAMTS13 [constitutively active a disintegrin and metalloproteinase with a thrombospondin type 1 motif, member 13]) is the subject of Great Britain (GB) patent application 2102208.2 (A novel biologic for treatment of thrombotic indications), International Patent Cooperation Treaty (PCT) application WO 2022/175666 published on August 25, 2022, Canadian patent application CA3208576 filed on August 25, 2022, European patent application 4294918 filed on December 27, 2023, Chinese patent application 202280015448.3 filed on October 24, 2023, US patent application 18/277,434 filed on February 17, 2022, South Korean patent application 10-2023-7031499 filed on September 14, 2023, Australian patent application 2022223366 filed on September 7, 2023, Singapore patent application 11202306167R filed on February 17, 2022, and Japanese patent application 2023-549646 filed on February 17, 2022.

### Supplemental Material

Supplemental Methods

Table S1

Figures S1–S2

ARRIVE Checklist

References 32,33
